# Supplementary material for: PLK-1 promotes the merger of the parental genome into a single nucleus by triggering lamina disassembly
Source: eLife. 2020 Oct 8;9:e59510. doi: 10.7554/eLife.59510 (PMC7544505; doi:10.7554/eLife.59510)
Supplement: Supplementary file 3. [file elife-59510-supp3.docx]

**VERTEBRATES**

LA_M Lamin A *Mus musculus* gi:112378771 gb:ABI16251.1

LC_M Lamin C *Mus musculus* gi:112378773 gb:ABI16252.1

LA_H Lamin A *Homo sapiens* gi:27436946 ref:NP_733821.1

LC_H Lamin C *Homo sapiens* gi:5031875 ref:NP_005563.1

LA_G Lamin A *Gallus gallus* gi:45384214 ref:NP_990618.1

LA-X Lamin A *Xenopus laevis* gi:156119433 ref:NP_001095210.1

LA_F Lamin A *Danio rerio* gi:190337691 gb:AAI63807.1

LB1_M Lamin B1 *Mus musculus* gi:188219589 ref:NP_034851.2

LB1_H Lamin B1 *Homo sapiens* gi:5031877 ref:NP_005564.1

LB1_G Lamin B1 *Gallus gallus* gi:45384220 ref:NP_990617.1

LB1_X Lamin B1 *Xenopus laevis* gi:147904084 ref:NP_001080053.1

LB1_F Lamin B1 *Danio rerio* gi:40254675 ref:NP_694504.2

LB2_F Lamin B2 *Danio rerio* gi:366392938 ref:NP_571077.2

LB2_X Lamin B2 *Xenopus laevis* gi:147901703 ref:NP_001080947.1

LB2_M Lamin B2 *Mus musculus* gi:113195686 ref:NP_034852.2

LB2_H Lamin B2 *Homo sapiens* gi:388240801 ref:NP_116126.3

LB2_G Lamin B2 *Gallus gallus* gi:45384202 ref:NP_990616.1

LL3_X Lamin L3 *Xenopus laevis* gi:148236667 ref:NP_001081545.1

LL3_F Lamin L3 *Danio rerio* gi:42476244 ref:NP_694505.2

**DROSOPHILA**

LC_D Lamin C *D. melanogaster* gi:442623692 ref:NP_001260974.1

LDm_D Lamin Dm *D. melanogaster* gi:17136290 ref:NP_476616.1

# NEMATODES

# Cb LMN-1 *C. briggsae* GenBank: CAP31352.2

# Cn LMN-1 C*. nigoni* GenBank: PIC51869

# Cbre LMN-1 *C. brenneri* GenBank: EGT38006.1

# Cre-LMN-1 *C. remanei* XP_00311226.1

# Ce LMN-1 *C. elegans* gi:17506429 ref:NP_492371.1
