# Supplementary material for: PLK-1 promotes the merger of the parental genome into a single nucleus by triggering lamina disassembly
Source: eLife. 2020 Oct 8;9:e59510. doi: 10.7554/eLife.59510 (PMC7544505; doi:10.7554/eLife.59510)
Supplement: Supplementary file 4. [file elife-59510-supp4.docx]

| **Designation** | **Sequence 5’-3’** | **Identifier** |
| --- | --- | --- |
| LMN-1 [H] (aa1-47) in pDONR201 | GGGGACAAGTTTGTACAAAAAAGCAGGCTTCATGTCATCTCGTAAAGGTACTCGTAGTTCTCG | OLP2148 |
| LMN-1 [H] (aa1-47) in pDONR201 | GGGGACCACTTTGTACAAGAAAGCTGGGTCTTAATGATCTTTCTCTTGAAGACGTGAAGTTTCTAGAAGCGTTGAGCC | OLP2164 |
| LMN-1 [H] (aa1-47) 3A S18A S35A T36A in pDONR201 | GGGGACAAGTTTGTACAAAAAAGCAGGCTTCATGTCATCTCGTAAAGGTACTCGTAGTTCTCG | OLP2148 |
| LMN-1 [H] (aa1-47) 3A S18A S35A T36A in pDONR201 | GGGGACCACTTTGTACAAGAAAGCTGGGTCTTAATGATCTTTCTCTTGAAGACGTGAAGTTTCTAGAAG | OLP2268 |
| LMN-1 [H] (aa1-47) 4A S21A S22A S24A S32A in pDONR201 | GGGGACCACTTTGTACAAGAAAGCTGGGTCTTAAAGACGTGAAGTTTCTAGAAGCGTTGAGCCAAATGAATCGTCGCCTCCTCC | OLP2177 |
| LMN-1 [H] (aa1-47) 4A S21A S22A S24A S32A in pDONR201 | GGGGACCACTTTGTACAAGAAAGCTGGGTCTTAAAGACGTGAAGTTTCTAGAAGCGTTGAGCCAAAAGCATCGTCGCCTCCTCC | OLP2178 |
| LMN-1 [H] (aa1-47) 7A S18A S21A S22A S24A S32A S35A T36A in pDONR201 | GGGGACAAGTTTGTACAAAAAAGCAGGCTTCATGTCATCTCGTAAAGGTACTCGTAGTTCTCG | OLP2148 |
| LMN-1 [H] (aa1-47) 7A S18A S21A S22A S24A S32A S35A T36A in pDONR201 | GGGGACCACTTTGTACAAGAAAGCTGGGTCTTAATGATCTTTCTCTTGAAGACGTGAAGTTTCTAGAAG | OLP2268 |
| LMN-1 [H] (aa1-47) 9A S18A S21A S22A S24A S32A S35A T36A T40A S41A in pDONR201 | GGCGACGATGCTTTTGGCGCAGCGCTTCTAGAAGCTGCACGTCTTCAAGAGAAAGATCATTAAGACCCAGC | OLP2493 |
| LMN-1 [H] (aa1-47) 9A S18A S21A S22A S24A S32A S35A T36A T40A S41A in pDONR201 | GCTGGGTCTTAATGATCTTTCTCTTGAAGACGTGCAGCTTCTAGAAGCGCTGCGCCAAAAGCATCGTCGCC | OLP2494 |
| LMN-1 [T] (aa387-548) in pDONR201 | GGGGACAAGTTTGTACAAAAAAGCAGGCTTCCTCAATCTTACTCAGGAGGCGCCAC | OLP2150 |
| LMN-1 [T] (aa387-548) in pDONR201 | GGGGACCACTTTGTACAAGAAAGCTGGGTCTTACATGATGGAACAACGATCGGCTGGGTCCG | OLP2151 |
| LMN-1 [T1] (aa387-436) in pDONR201 | GGGGACAAGTTTGTACAAAAAAGCAGGCTTCCTCAATCTTACTCAGGAGGCGCCAC | OLP2150 |
| LMN-1 [T1] (aa387-436) in pDONR201 | GGGGACCACTTTGTACAAGAAAGCTGGGTCTTAGCGACGGTTGAGATAATCAAT | OLP2234 |
| LMN-1 [T1] 4A (aa387-436) T397A S398A S403A S405A in pDONR201 | GGGGACAAGTTTGTACAAAAAAGCAGGCTTCCTCAATCTTACTCAGGAGGCGCCAC | OLP2150 |
| LMN-1 [T1] 4A (aa387-436) T397A S398A S403A S405A in pDONR201 | GGGGACCACTTTGTACAAGAAAGCTGGGTCTTAGCGACGGTTGAGATAATCAAT | OLP2234 |
| LMN-1 [T1] 6A (aa387-436) T390A, T397A, S398A, S403A, S405A, S406A in pDONR201 | GGGGACAAGTTTGTACAAAAAAGCAGGCTTCCTCAATCTTGCTCAGGAGGCGCCAC | OLP2269 |
| LMN-1 [T1] 6A (aa387-436) T390A, T397A, S398A, S403A, S405A, S406A in pDONR201 | GGGGACCACTTTGTACAAGAAAGCTGGGTCTTAGCGACGGTTGAGATAATCAAT | OLP2234 |
| Lamin Dm0 [H] (aa1-57) in pDONR201 | GGGGACAAGTTTGTACAAAAAAGCAGGCTTCATGTCGAGCAAATCCCGACGTGCTGG | OLP2283 |
| Lamin Dm0 [H] (aa1-57) in pDONR201 | GGGGACCACTTTGTACAAGAAAGCTGGGTCTTACTCCACCTTCTCGGCCACGCGCG | OLP2284 |
| Lamin Dm0 [H] (aa1-57) 4A T12A T20A S42A S44A in pDONR201 | GGGGACAAGTTTGTACAAAAAAGCAGGCTTCATGTCGAGCAAATCCCGACGTGCTGG | OLP2283 |
| Lamin Dm0 [H] (aa1-57) 4A T12A T20A S42A S44A in pDONR201 | GGGGACCACTTTGTACAAGAAAGCTGGGTCTTACTCCACCTTCTCGGCCACGCGCG | OLP2284 |
| Lamin A/C [H] (aa1-34) in pDONR201 | GGGGACAAGTTTGTACAAAAAAGCAGGCTTCATGGAGACCCCGTCCCAGCGGCGCG | OLP2288 |
| Lamin A/C [H] (aa1-34) in pDONR201 | GGGGACCACTTTGTACAAGAAAGCTGGGTCTTAGTCCTCCTTCTCCTGCAGCCGG | OLP2289 |
| Mutagenesis of Lamin A/C [H] (aa1-34)1A T18A | AGCGGGGCGCAGGCCAGCTCCGCTCCGCTGTCGCCCACCCGCATC | OLP2362 |
| Mutagenesis of Lamin A/C [H] (aa1-34)1A T18A | GATGCGGGTGGGCGACAGCGGAGCGGAGCTGGCCTGCGCCCCGCT | OLP2363 |
| LMN-1 in pET28b | AGGGCGGCCGCATCTGGTGGTGGTGGTGGTGAAAACCTGTATTTTCAGGGCGCCATGGATCCGGAGTTTATGTCATCTCG TAAAGGTACTCGT | OLP2199 |
| LMN-1 in pET28b | TCCCTCGAGTTACATGATGGAACAACGATCGGCT | OLP2200 |
